# The Structural Differences between a Glycoprotein Specific F-Box Protein Fbs1 and Its Homologous Protein FBG3

**DOI:** 10.1371/journal.pone.0140366

**Published:** 2015-10-13

**Authors:** Taichi Kumanomidou, Kazuya Nishio, Kenji Takagi, Tomomi Nakagawa, Atsuo Suzuki, Takashi Yamane, Fuminori Tokunaga, Kazuhiro Iwai, Arisa Murakami, Yukiko Yoshida, Keiji Tanaka, Tsunehiro Mizushima

**Affiliations:** 1 Department of Biotechnology, Graduate School of Engineering, Nagoya University, Chikusa-ku, Nagoya, Japan; 2 Picobiology Institute, Department of Life Science, Graduate School of Life Science, University of Hyogo, Kouto, Kamigori-cho, Ako-gun, Hyogo, Japan; 3 Laboratory of Molecular Cell Biology, Institute for Molecular and Cellular Regulation, Gunma University, Maebashi, Gunma, Japan; 4 Graduate School of Medicine, Kyoto University, Yoshida-Konoe-cho, Sakyo-ku, Kyoto, Japan; 5 Laboratory of Protein Metabolism, Tokyo Metropolitan Institute of Medical Science, Kamikitazawa, Setagaya-ku, Tokyo, Japan; 6 Ubiquitin Project, Tokyo Metropolitan Institute of Medical Science, Kamikitazawa, Setagaya-ku, Tokyo, Japan; University of Copenhagen, DENMARK

## Abstract

The *S*kp1-*C*ul1-*F*-box protein (SCF) complex catalyzes protein ubiquitination in diverse cellular processes and is one of the best-characterized ubiquitin ligases. F-box proteins determine the substrate specificities of SCF ubiquitin ligases. Among these, Fbs1/FBG1/FBXO2, Fbs2/FBG2/FBXO6, and Fbs3/FBG5/FBXO27 recognize the *N*-glycans of glycoproteins, whereas FBG3/FBXO44 has no sugar-binding activity, despite the high sequence homology and conservation of the residues necessary for oligosaccharide binding between Fbs1–3 and FBG3. Here we determined the crystal structure of the Skp1–FBG3 complex at a resolution of 2.6 Å. The substrate-binding domain of FBG3 is composed of a 10-stranded antiparallel β-sandwich with three helices. Although the overall structure of FBG3 is similar to that of Fbs1, the residues that form the Fbs1 carbohydrate-binding pocket failed to be superposed with the corresponding residues of FBG3. Structure-based mutational analysis shows that distinct hydrogen bond networks of four FBG3 loops, i.e., β2-β3, β5-β6, β7-β8, and β9-β10, prevent the formation of the carbohydrate-binding pocket shown in Fbs1.

## Introduction

The ubiquitin–proteasome system regulates many cellular processes, including signaling, cell cycle progression, apoptosis, immune and inflammatory responses, and protein quality control [1]. Protein ubiquitination is catalyzed by a cascade of reactions involving three types of enzymes such as the ubiquitin-activating (E1), ubiquitin-conjugating (E2), and ubiquitin-ligating (E3) enzymes. After several repetitions of the three-enzyme cascade, a polyubiquitin chain that is identified by the proteasome is formed, and subsequently the targeted protein is degraded into small peptides [2]. Among these enzymes, E3 enzymes are responsible for the selection of the target proteins. The SCF complex, one of the best characterized E3 enzymes, contains four subunits: Skp1, Cul1, RING-finger protein (Rbx1), and F-box protein. F-box proteins provide substrate specificity to the SCF complex [3], and consist of the common F-box domain interacting with Skp1, a linker region, and a substrate-binding domain (SBD). Structurally, SBD is classified into three classes: FBXW, which contains WD-40 domains; FBXL, which contains leucine-rich repeats; and FBXO, which does not contain any of these domains. Fbs1/FBG1/FBXO2, an FBXO family protein, recognizes high-mannose oligosaccharide modifications [4] and belongs to a subfamily consisting of at least five homologous proteins [5,6]. Among these, Fbs1, Fbs2/FBG2/FBXO6, and Fbs3/FBG5/FBXO27 recognize high-mannose oligosaccharides [7–9]. Fbs proteins recognize aberrant *N*-linked glycoproteins for the endoplasmic reticulum (ER)-associated degradation system, resulting in degradation by the proteasome. Crystal structures of SBD of Fbs1, Skp1–Fbs1 and SBD-RNase B have been reported [10,11]. These crystal structures revealed that the structures of SBD are identical with and without substrate, that SCFFbs1 recognizes the innermost Man3GlcNAc2 in *N*-glycans, and that the exposure of the innermost position of *N*-glycans serves as a signal for Fbs1 to recognize denatured glycoproteins.

**Fig 1:**
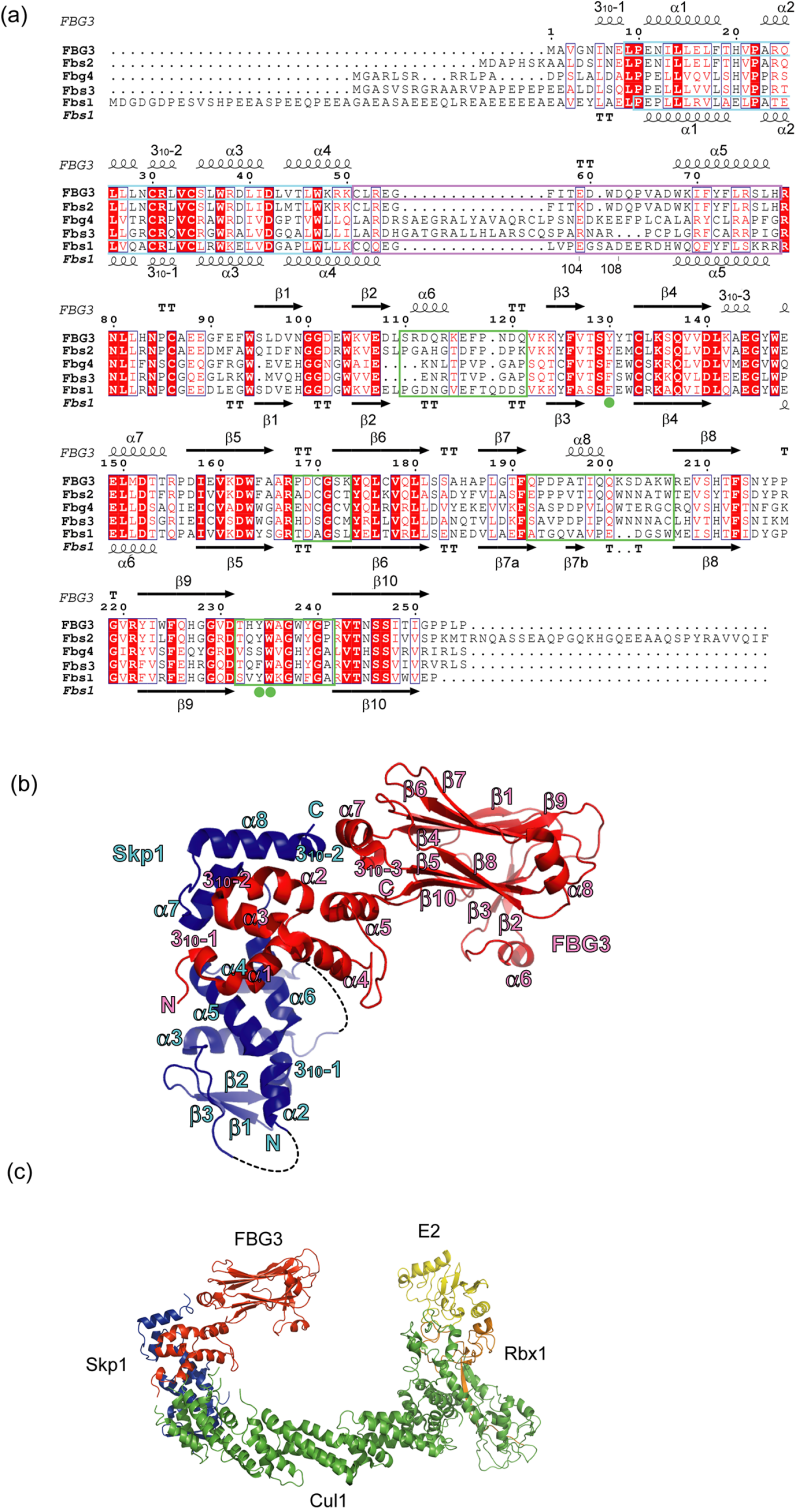
Structure of the Skp1–FBG3 complex. (A) Sequence conservation and secondary structure elements of Fbs family proteins. The alignment was generated by ClustalW [25]; α-helices are depicted as coils, β-strands as arrows, and turns by the letters TT. Conserved residues are boxed in white on a red background and similar residues are boxed in red on a white background. F-box and linker domains are boxed in cyan and purple, respectively. β2-β3, β5-β6, β7-β8, and β9-β10 loops are indicated by green boxes. Filled green circles indicate residues forming the carbohydrate-binding pocket. The figure was generated by ESPript [26]. (B) Overall structure of the complex. Skp1 and FBG3 are colored blue and red, respectively. The secondary structure elements for Skp1 and FBG3 are labeled in blue and red, respectively. Dotted lines represent disordered regions.(C) Model of the SCF^FBG3^ complex bound to E2. Cul1, Rbx1, Skp1, FBG3, and E2 are colored green, orange, blue, red, and yellow, respectively. A model of SCF^FBG3^ was simply constructed by superposition of the Skp1 subunits from the Skp1–FBG3, Skp1–Fbs1, and Skp1–Cul1–Rbx1 structures [27] (PDB ID code 1LDK), the RING-finger domains derived from Rbx1 and the c-Cbl subunit of the c-Cbl-UbcH7 structure [28] (PDB ID code 1FBV), and the E2 subunits of the c-Cbl-UbcH7 structure, using the program LSQKAB [29].

The sugar-binding activity of FBG3/FBXO44 has not been detected [7,8], although SBD in FBG3 has a 52%, 68%, and 43% sequence identity to that in Fbs1, Fbs2, and Fbs3, respectively. In addition, the residues necessary for binding to *N*-glycans in Fbs1, Fbs2, and Fbs3 are conserved in FBG3 (Fig 1A). Recently, it has been reported that FBG3 mediates nonglycoprotein BRCA1 ubiquitination [12]. To understand the mechanistic details of sugar recognition in Fbs proteins, we determined the crystal structure of the Skp1–FBG3 complex and compared it with that of Fbs1.

## Materials and Methods

### Cloning, expression and purification of Fbs proteins

The Skp1–FBG3 complex was used for structure determination and *in vitro* carbohydrate binding assay. Cloning, expression, and purification of recombinant Skp1–FBG3 protein complex were described previously [13]. Briefly, 6xHis-tagged human Skp1 and FBG3 in pET28b plasmid were expressed in *E*. *coli* BL21 (DE3). The recombinant protein complex was purified in a stepwise process using Ni-NTA affinity, anion-exchange, and gel-filtration chromatography. For structure determination, the 6xHis-tag region was removed from Skp1 by treatment with PreScission protease (GE Healthcare) after the Ni-NTA step. The codon-optimized FBG3 loop mutants for expression in *E*. *coli* were designed by GeneArt (Life Technologies), and the synthesized DNA was cloned into a pET21a vector. Each FBG3 loop mutant in pET21a and Skp1 in pET28b were co-transformed into *E*. *coli* BL21 (DE3). The expressed Skp1–FBG3 loop mutants were purified by the same purification procedure used for the wild-type Skp1–FBG3 protein.

The SBD of Fbs1 (117–297) and Skp1–Fbs1 complex was used for *in vitro* carbohydrate binding assay. Cloning, expression, and purification of recombinant wild-type Fbs1 SBD was described previously [10]. Briefly, 6xHis-tagged SBD of murine Fbs1 in pET15b plasmid was expressed in *E*. *coli* Rosetta (DE3). Recombinant protein was purified in a stepwise process using Ni-NTA affinity and gel-filtration chromatography. The Fbs1 SBD mutants were constructed by inverse PCR-based KOD-Plus Mutagenesis kit (Toyobo). The codon-optimized Fbs1 SBD loop mutants were designed and synthesized by GeneArt, and the DNA fragments were subcloned into pET15b plasmid. The expressed Fbs1 SBD mutants were purified using the same procedure used for the wild-type. Cloning, expression, and purification of recombinant Skp1–Fbs1 complex was described previously [11]. Briefly, 6xHis-tagged human Skp1 and murine Fbs1 in pET28b plasmid were expressed in *E*. *coli* Rosetta (DE3). Recombinant protein was purified using the same procedure used for the Skp1–FBG3 complex.

### Crystallization, data collection, and structure determination of Skp1–FBG3 complex

Crystallization and preliminary X-ray diffraction analysis of the Skp1–FBG3 complex were described previously [13]. Diffraction data for Skp1–FBG3 were collected at 100 K on beamline BL44XU at SPring-8 (Hyogo, Japan). Data sets were indexed using DENZO and scaling was performed using SCALEPACK [14]. The structure was solved by molecular replacement using CCP4/MOLREP [15,16] with the Skp1–Fbs1 complex (PDB ID code 2E31) as a search model. Model-building and refinement were conducted with COOT [17] and REFMAC5 [18], respectively. The final refined model contained residues 2–253 of FBG3 and 1–32, 42–69, and 84–162 of Skp1. The data collection and final refinement statistics are summarized in Table 1.

**Table 1:** Data collection and refinement statistics.

**Data collection**	
Space group	*P*2_1_2_1_2_1_
Unit cell parameters *a*, *b*, *c* (Å)	34.1, 76.6, 193.9
Wavelength (Å)	0.9
Resolution (Å)	97.1–2.6 (2.65–2.6)*
No. of observations	77,996
No. of unique reflections	16,474
Completeness (%)	99.5 (99.6)
*<I*/σ(*I*)>	19.8 (6.3)
Redundancy	4.8 (4.6)
*R*_merge_ (%)	6.6 (30.8)
**Refinement**	
Resolution range (Å)	60.1–2.6 (2.67–2.6)
No. of reflections in working set	15516 (1076)
No. of reflections in test set	826 (52)
*R*_work_ (%)	20.5 (28.2)
*R*_free_ (%)	26.6 (44.4)
Protein atoms	3,221
Solvent	16
R.m.s.d of bond length (Å)	0.013
R.m.s.d of bond angle (°)	1.6
Ramachandran analysis Preferred / Allowed / Outlier (%)	93.7 / 5.7 / 0.5

* Data for the outer shell are in parentheses. *R*_merge_ = Σ_hkl_Σ_i_ |*I*(*hkl*)-<*I*(*hkl*)>|/Σ_hkl_Σ_i_(*hkl*). *R*_work_ = Σ||*F*_o_|-|*F*_c_||/|*F*_o_|. *R*_free_ was calculated using 5.0% of the data (test set) that was not used in structure refinement.

### *In vitro* pull-down carbohydrate-binding assay

For the *in vitro* pull-down carbohydrate-binding assay of Fbs1, 15 μg of purified Fbs1 SBDs were incubated with 30 μL of Ni-NTA agarose resin, and the resin with immobilized proteins was washed three times with 600 μL of pull-down buffer (20 mM Tris-HCl pH 7.5, 20 mM imidazole, and 500 mM NaCl). The resulting immobilized His-tagged SBDs were incubated with 10.7 μg of ribonuclease B (RNase B from bovine pancreas; Sigma Aldrich) at 4°C for 1 h, washed three times, and suspended in 20 μL of SDS-PAGE sample buffer (4% SDS, 500 mM 2-ME, 8% glycerol, 80 mM Tris-HCl pH 6.8, and 0.02% bromophenol blue). The resultant proteins were subjected to SDS-PAGE, followed by Coomassie brilliant blue (CBB) staining. The intensity of each CBB-stained band was analyzed by densitometry, using the “Gel Submenu” of ImageJ software (National Institutes of Health). The peak profiles were plotted using intensity of bands. The relative quantities of recombinant proteins and RNase B were estimated from the area size of the peak profiles. RNase B binding activities were estimated using the peak area size ratio of each band (RNase B/recombinant F-box protein). The activity of mutants was normalized against the control (RNase B/Fbs1 wild type as 100%). These measurements were repeated at least three times and statistical results were calculated using Excel (Microsoft). The activities were represented as the mean ± S.E. The unpaired Student’s *t* test was used for analyzing differences between the control (wild type) and mutants, or notable mutant pair, as indicated by the guide (***, *p* < 0.001; **, *p* < 0.01; *, *p* < 0.05; *n* = 3).

The SBD of FBG3 has a tendency to aggregate. Such aggregation could be suppressed by co-expression with Skp1. The assay procedure for Skp1–FBG3 was almost identical to that of Fbs1 SBD. Briefly, purified FBG3 with His-tagged Skp1 was immobilized on Ni-NTA agarose resin and incubated with RNase B. The bound Skp1–FBG3 and RNase B were analyzed using SDS-PAGE with CBB staining. Further, 20 μg of purified Skp1-wild-type FBG3 and 6.2 μg of RNase B were used for pull-down assay.

## Results and Discussion

### Structure of the Skp1–FBG3 complex

The structure of the human Skp1–FBG3 complex was determined by the molecular replacement method at a resolution of 2.6 Å (Table 1). The crystal belongs to the space group *P*2_1_2_1_2_1_, with a single copy of the Skp1–FBG3 complex in the asymmetric unit. The FBG3 structure consists of an N-terminal 3_10_ helix (3_10_−1), an F-box domain (residues 9–50), a linker domain (residues 51–78), and SBD (residues 79–255; Fig 1A and 1B). The F-box domain of FBG3 contains the same four α-helix structures (α1–4) observed in the domain of Fbs1 (0.67 Å r.m.s. deviation for 42 Cα atoms). The linker domain of FBG3 consists of a loop structure (linker loop; residues 51–67) and the α5 helix (residues 68–77). SBD is composed of a 10-stranded antiparallel β-sandwich (β1–β10), with three α-helices (α6, α7, and α8) and one 3_10_-helix (3_10_−3). Although the overall structure of the SBD of FBG3 resembles that of Fbs1, FBG3 has two additional α-helices (α6 and α8) and one 3_10_-helix (3_10_−3). The α6, α8, and 3_10_−3 helices are located between β2 and β3, β7 and β8, and β4 and α7, respectively.

The overall spatial arrangement of Skp1 and FBG3 is highly analogous to those of previously reported Skp1–F-box protein complexes, e.g., Skp1–Skp2 [19], Skp1–Cdc4 [20], Skp1–β-TrCP1[21], Skp1–Fbx4 [22], and Skp1–Fbxl3-CRY [23]. However, the position of two Skp1 helices (H7 and H8) in SCF^Fbs1^ differs from that of the conventional SCF complex (S1 and S2 Figs). In the SCF^FBG3^ model, each subunit is arranged in a manner similar to that of other F-box proteins, and the distance between the E2 active-site cysteine and the tip of the SBD is approximately 60 Å, which is similar to the value that was reported previously [24] (Fig 1C). This structure may allow for the ubiquitination of substrates.

### Linker domain between the F-Box and SBD

**Fig 2:**
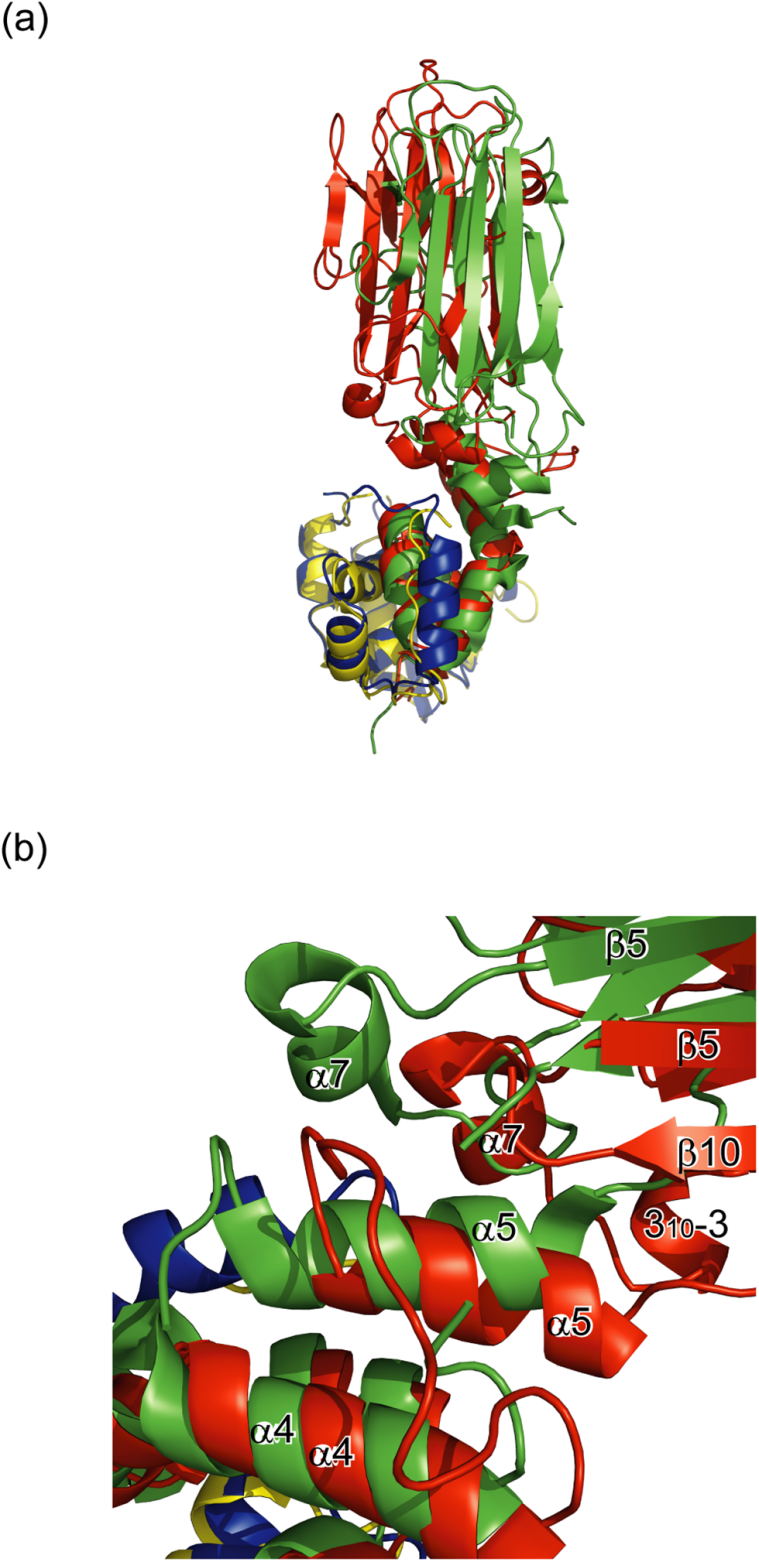
Comparison of the linker domains in Skp1–FBG3 and Skp1–Fbs1. (A) Comparison of the crystal structure of the Skp1 (blue)–FBG3 (red) complex and the Skp1 (yellow)–Fbs1 (green) complex. (B) Magnified view of the linker region of the substrate-binding domain.

The homology of amino acid sequences in the linker domains between Fbs1 and FBG3 is low compared with those between the F-box domain and SBD (Fig 1A). To examine the structural differences of their linker domains, we superposed these structures using the program LSQKAB (Fig 2A). The structures of FBG3 and Fbs1 linker domains closely resemble each other (r.m.s. deviation = 2.0 Å for the Cα atoms). The F-box domains are well aligned with each other and the α5 helix is tilted by approximately 10°. The 10° tilt angle of α5 causes the observed differences in the orientation of SBDs (Fig 2A and 2B). The helical structure (3_10_−3) is specifically found in the FBG3 loop between β4 and α7, beside the α5. The residues Leu141 and Glu144, located in 3_10_−3, form hydrogen bonds with Arg79, His83, and Arg221 (located in the α5-β1 and β8-β9 loops). These hydrogen bonds may affect the orientation of SBD. Moreover, although the structure of the linker loop in FBG3 was determined from the electron density map, the residues 104–108 of Fbs1 were disordered. The inefficient SCF complex formation of Fbs1 has been reported [30]. In contrast to Fbs1, Fbs2 and FBG3, whose linker domain sequences are identical (Fig 1A), form effective SCF complexes [7,8,30]. Thus, the linker domain may provide for the orientation of the linkage between the F-box and substrate-binding domains and the formation of the SCF complex.

### Comparison of SBD between FBG3 and Fbs1

**Fig 3:**
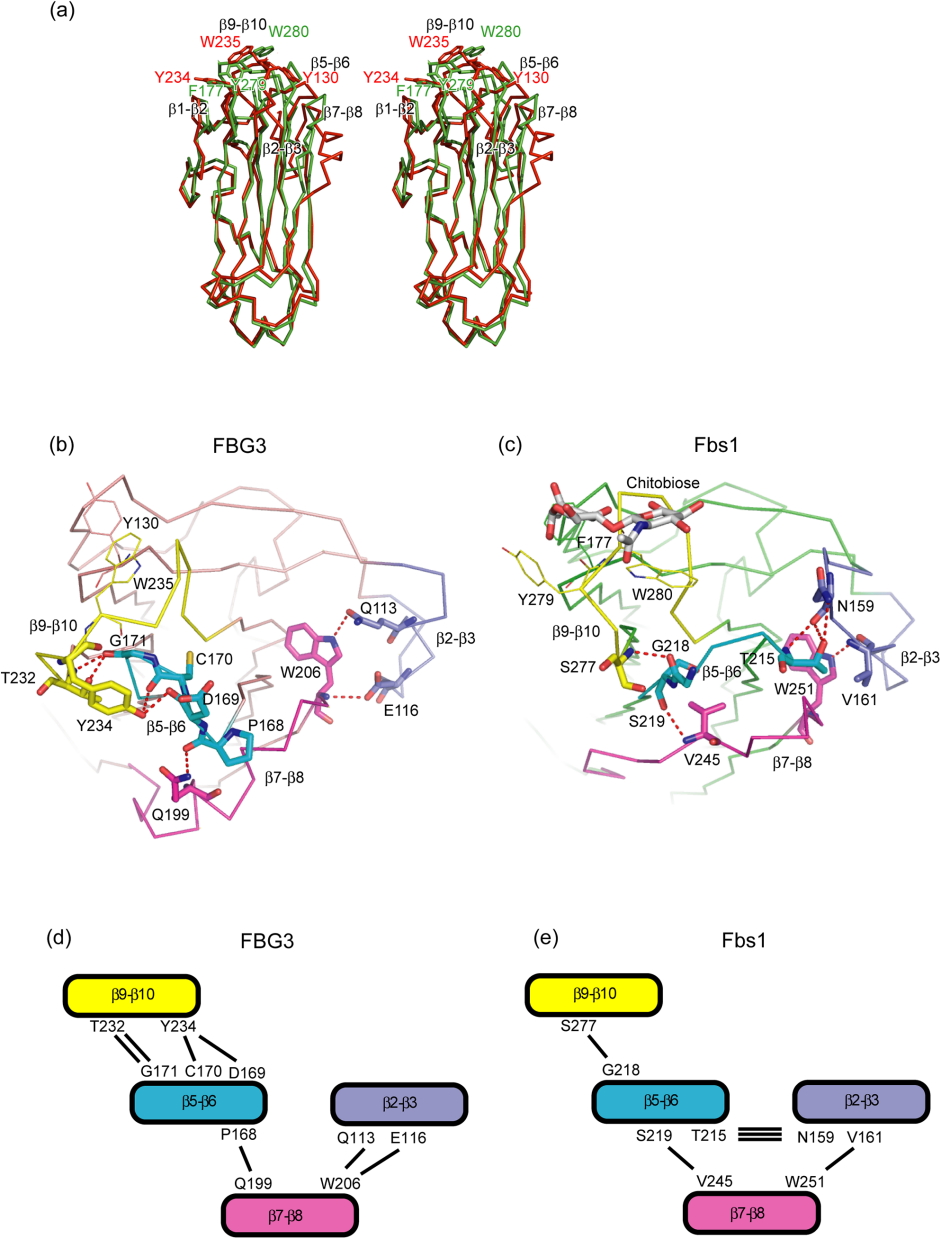
Comparison of the substrate-binding domain (SBD) between FBG3 and Fbs1. (A) Stereo view of the comparison between SBD of FBG3 (red) and SBD of Fbs1 (green). (B, C) Comparison of the intramolecular hydrogen bonds in the SBD of FBG3 (B) and SBD of Fbs1 (C). Hydrogen bonds are represented as dashed lines. The loops β2-β3, β5-β6, β7-β8, and β9-β10 are colored blue, cyan, magenta, and yellow, respectively. The residues of the hydrogen bonding pair are depicted as stick models. The carbohydrate-binding residues are depicted as line models. (D, E) The schematic view of the hydrogen bond networks between four loops in FBG3 (D) and Fbs1 (E). The loops β2-β3, β5-β6, β7-β8, and β9-β10 are labeled and colored as in (B, D). Hydrogen bonds are represented as solid lines. The residues of the hydrogen bonding pair are labeled beside each loop to which they belong.

SBD in the Skp1–FBG3 complex is composed of a 10-stranded antiparallel β-sandwich, and it can be superposed on SBD in the Skp1–Fbs1 complex with an average r.m.s. deviation of 2.1 Å for the Cα atoms (Fig 3A). The main differences among these SBDs are found in the site opposite to that of the Skp1-binding site. X-ray crystallographic and mutagenesis studies of Fbs1 have revealed that the hydrophobic interactions between GlcNAc–GlcNAc and Phe177, Tyr279, and Trp280 in Fbs1 are essential for binding to the *N*-glycan of glycoproteins [10]. In FBG3, the positions corresponding to Phe177, Tyr279, and Trp280 in Fbs1 are occupied by Tyr, Tyr, and, Trp, respectively (Fig 1A). However, the comparison of the substrate-binding pocket of Fbs1 (Phe177, located in the loop β3-β4, and Tyr279 and Trp280, located in the loop β9-β10) and the corresponding residues of FBG3 (Tyr130, Tyr234, and Trp235) are not well superposed (Fig 3A). The r.m.s. deviation values between FBG3 and Fbs1 for the groups of atoms comprising the main chain and all atoms are 1.95 Å and 5.19 Å, respectively. The side chains of Tyr234 and Trp235 in FBG3 are oriented in opposite directions to those of Tyr279 and Trp280 in Fbs1 (Fig 3B and 3C and S3 Fig). Although the sequence similarity between FBG3 and Fbs1 is high, i.e., 133 out of 255 residues in FBG3 are identical with those in Fbs1, four loops (β2-β3, β5-β6, β7-β8, and β9-β10) exhibit different conformations. Therefore, the differences in the loop conformation of β9-β10 in Fbs1 and FBG3 may affect the arrangement of Tyr130, Tyr234, Trp235, and Ala236 in FBG3. These conformational differences are supposedly caused by the distinct hydrogen bond networks among the loops β2-β3, β5-β6, β7-β8, and β9-β10. The hydrogen bond network in FBG3 is found between the loops β5-β6 (residues Asp169, Cys170, and Gly171) and β9-β10 (residues Thr232 and Tyr234; Fig 3B and 3D), whereas Fbs1 has only a single hydrogen bond between the loops β5-β6 (Gly218) and β9-β10 (Ser277; Fig 3C and 3E), suggesting that the interaction of these two loops in FBG3 is tighter than that in Fbs1. In contrast, the interaction between the loops β5-β6 and β2-β3 is tighter in Fbs1 than in FBG3. Compared with Fbs1, the loops β2-β3 and β7-β8 in FBG3 involve helical structures. The α6 and α8 helices are located at the top of the β-sandwich, and form hydrogen bonds between Gln199 in the loop α8-β8 and Pro168 in the loop β5-β6 (Figs 1A and 3B). These differences in the hydrogen bond networks among the loops β2-β3, β5-β6, β7-β8, and β9-β10 in FBG3 may avoid the formation of a substrate-binding pocket, as observed in Fbs1.

### Effects of Fbs1 mutations on substrate recognition

**Fig 4:**
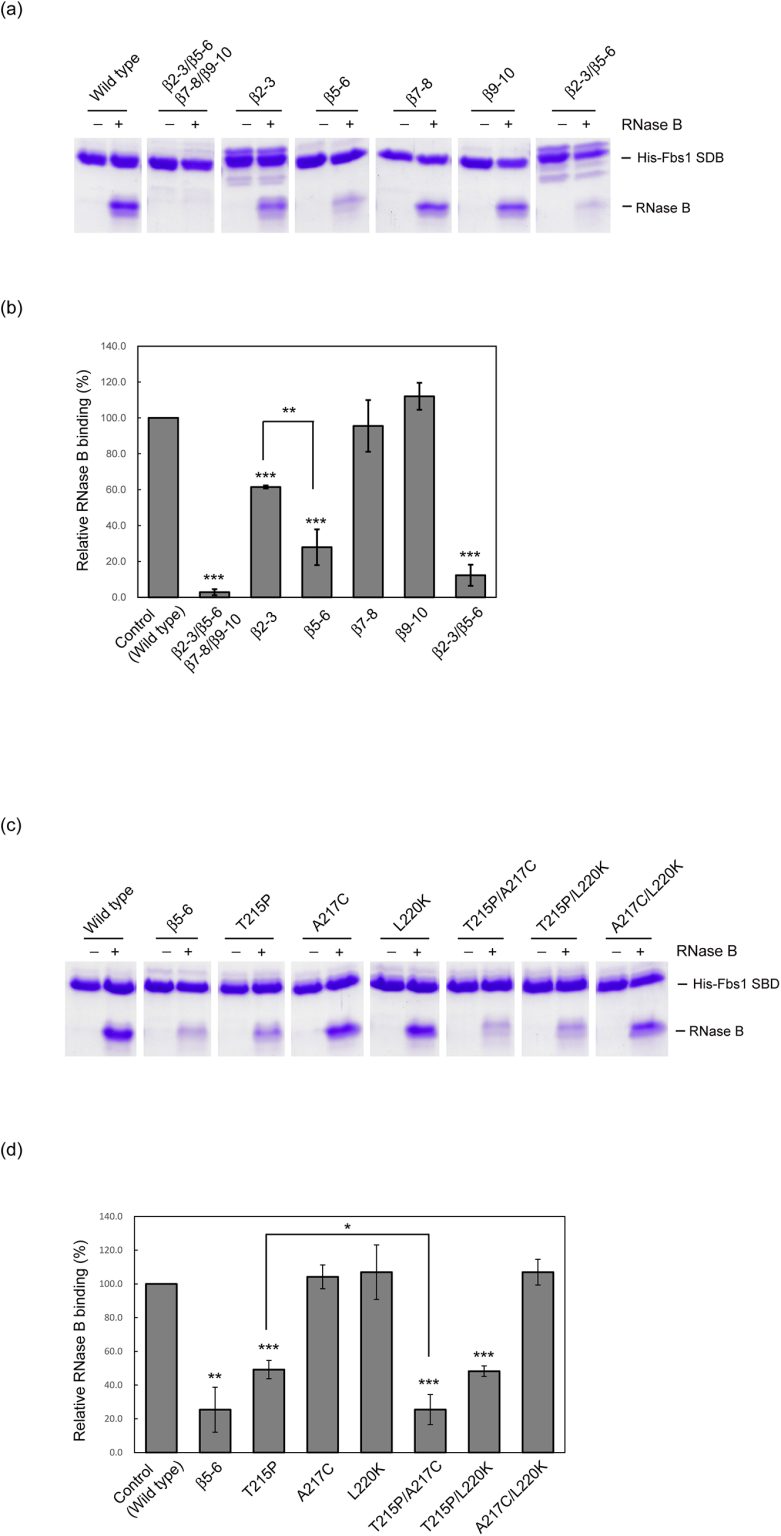
*In vitro* RNase B binding activities of the Fbs1 and its mutants using pull-down assay. (A) Characterization of the crucial loops in Fbs1 for carbohydrate binding. (B) Comparison of relative RNase B binding activities between wild type (control) and loop mutants shown in (A). Three independent pull-down assays were analyzed. Error bars represent means ± S.E. (C) Determination of pivotal residues in the loop β5-β6 for the carbohydrate-binding pocket formation. (D) Effects of mutations introduced in non-conserved residues in the loop β5-β6 shown in (C). Three independent pull-down assays were analyzed. Error bars represent means ± S.E.

To confirm that the formation of the substrate-binding pocket in Fbs1 provides optimum interactions among loops (Fig 3D and 3E), we simultaneously substituted the four loops β2-β3, β5-β6, β7-β8, and β9-β10 in Fbs1 with those of FBG3, and subsequently examined the *in vitro* activities in binding ribonuclease B (RNase B), which has a single high-mannose oligosaccharide. This mutant lost its binding capacity to RNase B, indicating that appropriate loop–loop interactions form the correct binding pocket (Fig 4A and 4B). Furthermore, we investigated whether the replacement of each loop affects the binding to *N*-glycan. The replacement of the loop β9-β10, involving residues that form the carbohydrate-binding pocket, had no effect on the binding activity, suggesting that the hydrogen bond between Gly218 and Ser277 is expendable for substrate binding. Although both the loops β2-β3 and β7-β8 in FBG3 contain helical structures and have less homology with the corresponding loops in Fbs1, the individual replacement of either loop β2-β3 or β7-β8 has little or no effect on the binding to RNase B. The replacement of the short loop β5-β6 reduced the binding, suggesting that the loop β5-β6 is pivotal for substrate binding pocket formation. However, the residues forming hydrogen bond networks between the loops β5-β6 (Asp169 and Gly171) and β9-β10 (Thr232 and Tyr234) in FBG3 are conserved in Fbs1 (Fig 1A). Therefore, we introduced mutations in the nonconserved residues Thr215 and Ala217 in the loop β5-β6 and Leu220 at the beginning of β6 (Fig 4C and 4D). The A217C and L220K mutations had no effect on binding, whereas the T215P mutation reduced the binding activity. Thr215 in Fbs1 forms three hydrogen bonds with Asn159 in the loop β2-β3, suggesting that the tight interaction between the loops β2-β3 and β5-β6 is necessary for the formation of the carbohydrate-binding pocket in Fbs1. The replacement of the loops β2-β3 and β5-β6 dramatically reduced the binding, as did the substitution of four loops (Fig 4A and 4B). The two residue mutations had no obvious synergistic effect on the binding, excluding T215P/A217C (Fig 4C and 4D). Because Ala217 lies near the loop β9-β10 in Fbs1, the A217C mutation may cause steric hindrance between the loops β5-β6 and β9-β10 in the T215P/A217C mutant, in which the distance between these loops is decreased by the T215P mutation (S4 Fig).

### Effects of FBG3 mutations on substrate binding ability

**Fig 5:**
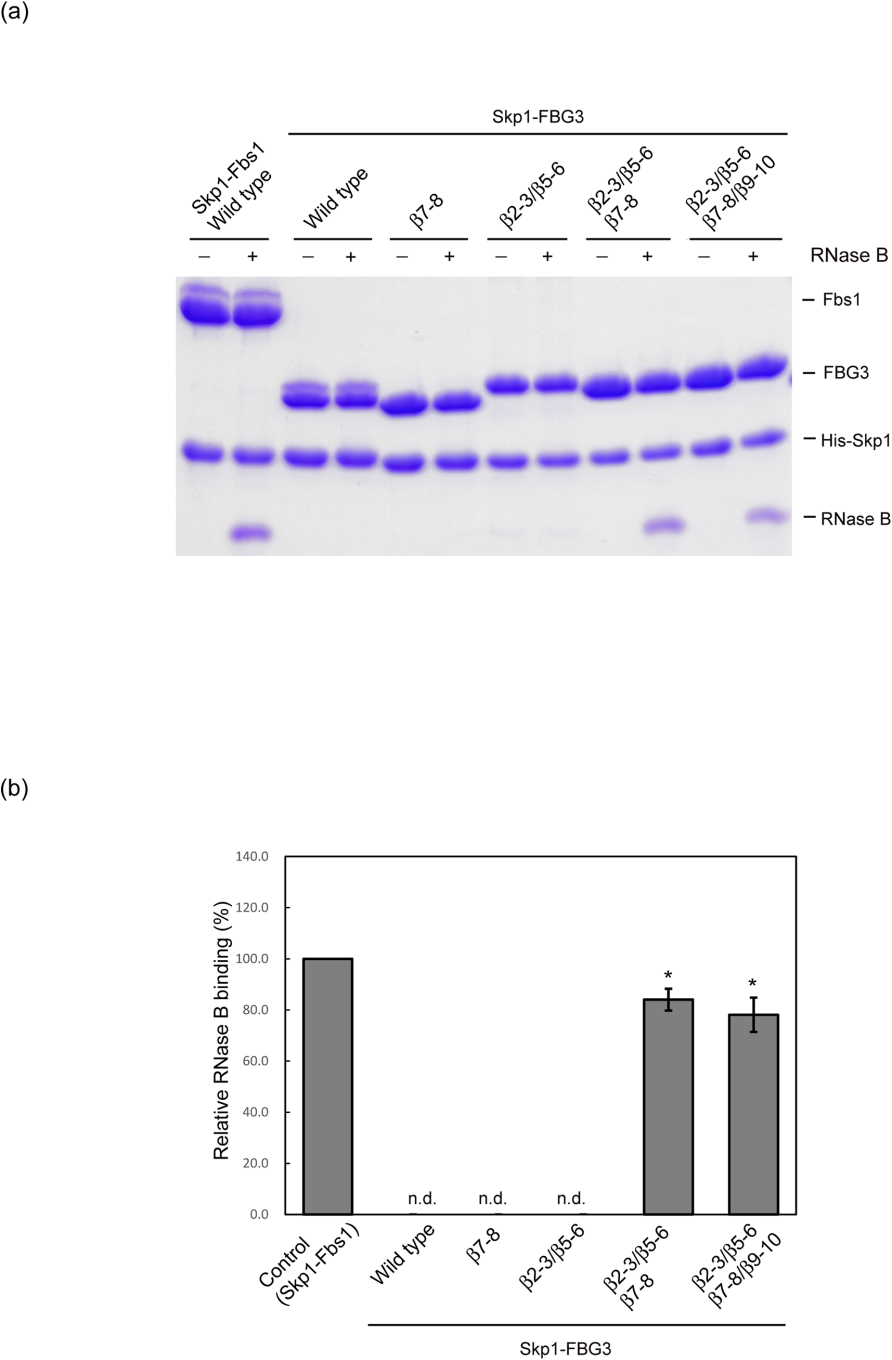
*In vitro* RNase B binding activities of the Skp1–FBG3 complex and its mutants using pull-down assay. (A) Determination of the crucial loops for the carbohydrate-binding pocket formation in FBG3. (B) Comparison of relative RNase B binding activities between Skp1–Fbs1 wild type (control) and Skp1–FBG3 loop mutants shown in (A). Three independent pull-down assays were analyzed. Error bars represent means ± S.E.

To confirm the contribution of the loops β2-β3, β5-β6, β7-β8, and β9-β10 in the formation of the substrate-binding pocket in Fbs1 directly, we simultaneously substituted the four loops β2-β3, β5-β6, β7-β8, and β9-β10 in FBG3 with those of Fbs1, and subsequently examined the *in vitro* activities in binding RNase B. This mutant could bind to RNase B (Fig 5A and 5B). Although the tight interactions between the loops β2-β3 and β5-β6 are likely to contribute to carbohydrate-binding, the replacement of these loops failed to show RNase B binding activity, suggesting that the hydrogen bond network between the β2-β3 and β5-β6 loops is not sufficient to attenuate the interaction between the β5-β6 and β9-β10 loops. Therefore, we substituted the β7-β8 loop in addition to the β2-β3 and β5-β6 loops. This mutant showed binding activity for RNase B. However, a single β7-β8 loop mutant did not show RNase B binding activity. These results indicate that the hydrogen bond networks among the β2-β3, β5-β6, and β7-β8 loops are necessary for the formation of the carbohydrate-binding pocket in the β9-β10 loop.

## Conclusions

In this study, we determined the crystal structure of the Skp1–FBG3 complex, at a resolution of 2.6 Å. Despite the high sequence and structural homology between Fbs1 and FBG3, no sugar-binding activity of FBG3 has been reported. We confirmed that FBG3 does not possess the carbohydrate-binding pocket that is observed in Fbs1. The carbohydrate-binding pocket is formed by the residues in the loops β3-β4 and β9-β10 in Fbs1, whereas distinct hydrogen-bond networks among the four loops β2-β3, β5-β6, β7-β8, and β9-β10 in FBG3 prevent it from forming the carbohydrate-binding pocket shown in Fbs1. Although the functions of FBG3 are unclear, our structural study of the Skp1–FBG3 complex provides a framework for future studies of the Fbs family SCF ubiquitin ligase.

## Accession numbers

The atomic coordinates and structure factors of Skp1–FBG3 have been deposited in the Protein Data Bank with accession number type PDB ID: 3WSO.

## Supporting Information

S1 Fig Model of the SCF^Fbs1^ complex bound to E2. Cul1, Rbx1, Skp1, Fbs1, E2, and RNase B are colored green, orange, blue, magenta, yellow, and cyan, respectively.
(TIF)

S2 Fig Structure of the Skp1-F-box protein complex. (A) Skp1 (blue)–FBG3 (red), (B) Skp1 (yellow)–Fbs1 (green) [11], (C) Skp1 (black)–Cdc4 (purple) [20], (D) Skp1 (black)–β-TrCP1 (cyan) [21], (E) Skp1 (black)–Fbx4 (pink) [22], and (F) Skp1 (black)–Fbxl3 (lime)-CRY (blue-purple) [23]. The two Skp1 helices in Skp1–FBG3 (H7 and 3_10_−2) are marked with dashed red circles.
(TIF)

S3 Fig Substrate-binding pocket of SBD. (A, B) Surface potential representation of the substrate-binding pocket of the SBD in FBG3 (A) and in Fbs1 (B). The bound Man_3_GlcNAc_2_ (cyan) and residues involved in the substrate binding (FBG3: magenta, Fbs1: green) are represented by a stick model. Surfaces are colored according to their electrostatic potential from red (negative) to blue (positive).
(TIF)

S4 Fig Stereo view of the substrate-binding sites of Fbs1. Fbs1 A217C model and FBG3 are light green and pink. Non-conserved amino acids at the loop β5-β6 are represented using stick models. Hydrogen bonds between non-conserved residues and other loops are indicated by dotted lines. A217C and V278 in Fbs1 are represented using sphere models. The sphere models show the side chain conflict between the sulfur atom (yellow sphere) of A217C model and carbon atom (green sphere) of V278.
(TIF)
