# Supplementary figures and images for: The Structural Differences between a Glycoprotein Specific F-Box Protein Fbs1 and Its Homologous Protein FBG3
Source: PLoS One. 2015 Oct 13;10(10):e0140366. doi: 10.1371/journal.pone.0140366 (PMC4603797; doi:10.1371/journal.pone.0140366)

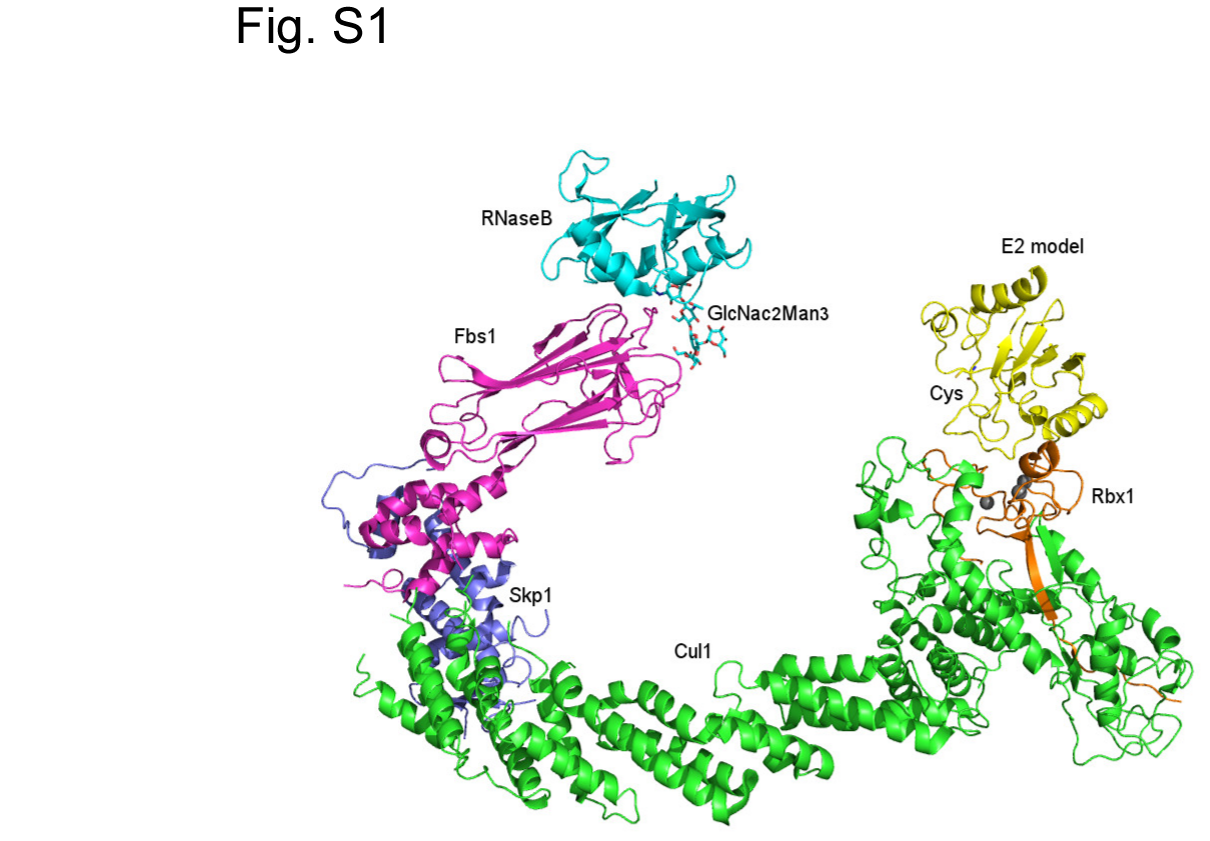

Supplement: S1 Fig — Cul1, Rbx1, Skp1, Fbs1, E2, and RNase B are colored green, orange, blue, magenta, yellow, and cyan, respectively. (TIF) [file pone.0140366.s001.tif]

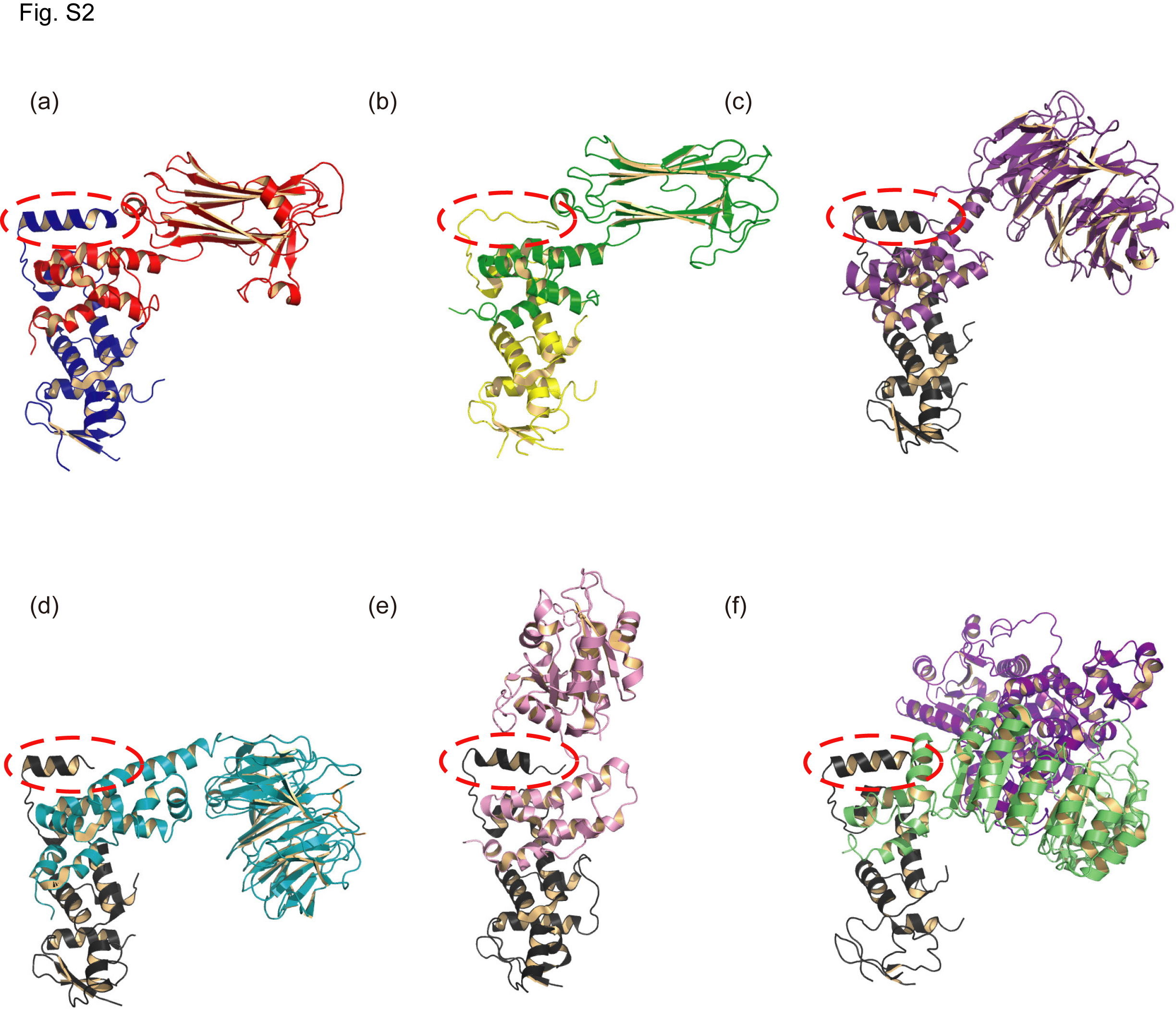

Supplement: S2 Fig — (A) Skp1 (blue)–FBG3 (red), (B) Skp1 (yellow)–Fbs1 (green) [11], (C) Skp1 (black)–Cdc4 (purple) [20], (D) Skp1 (black)–β-TrCP1 (cyan) [21], (E) Skp1 (black)–Fbx4 (pink) [22], and (F) Skp1 (black)–Fbxl3 (lime)-CRY (blue-purple) [23]. The two Skp1 helices in Skp1–FBG3 (H7 and 310−2) are marked with dashed red circles. (TIF) [file pone.0140366.s002.tif]

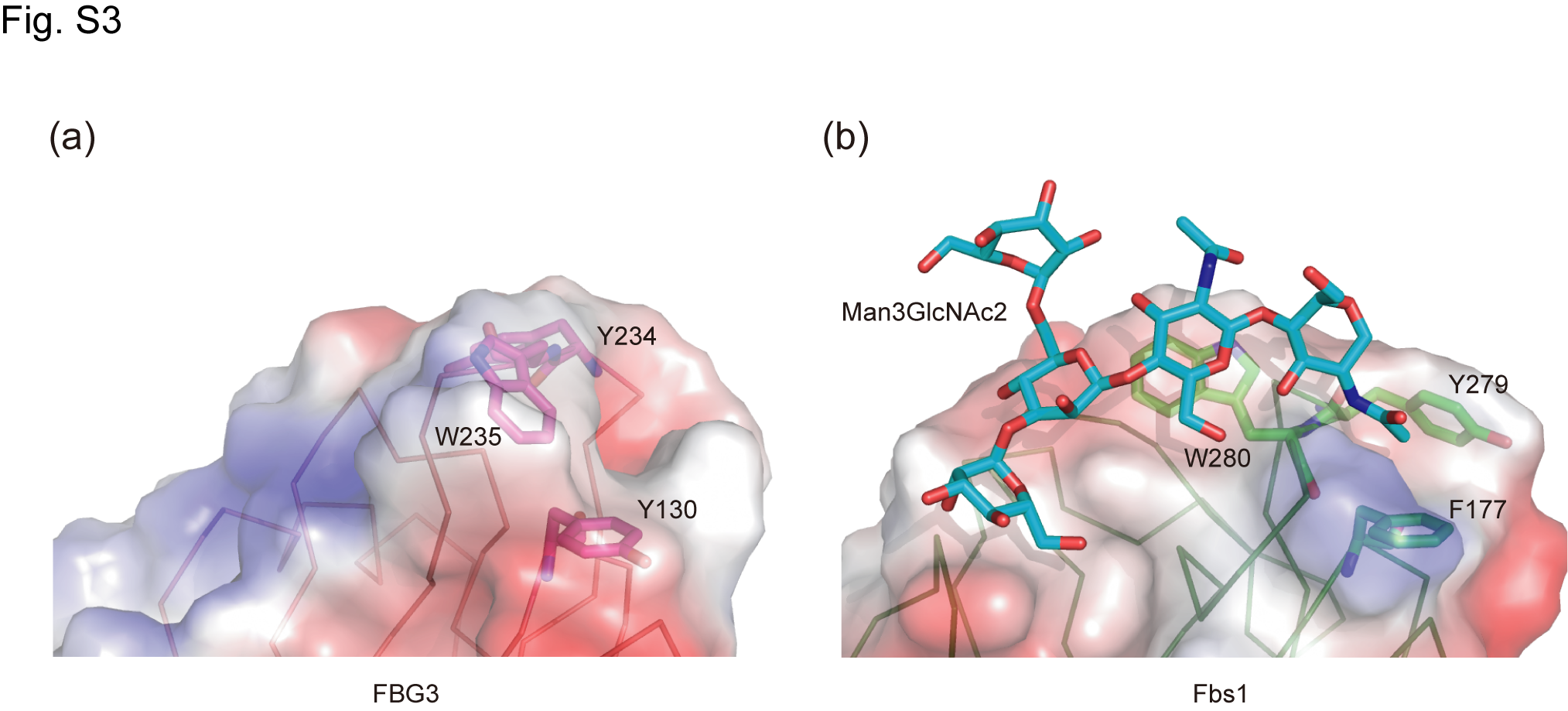

Supplement: S3 Fig — (A, B) Surface potential representation of the substrate-binding pocket of the SBD in FBG3 (A) and in Fbs1 (B). The bound Man3GlcNAc2 (cyan) and residues involved in the substrate binding (FBG3: magenta, Fbs1: green) are represented by a stick model. Surfaces are colored according to their electrostatic potential from red (negative) to blue (positive). (TIF) [file pone.0140366.s003.tif]

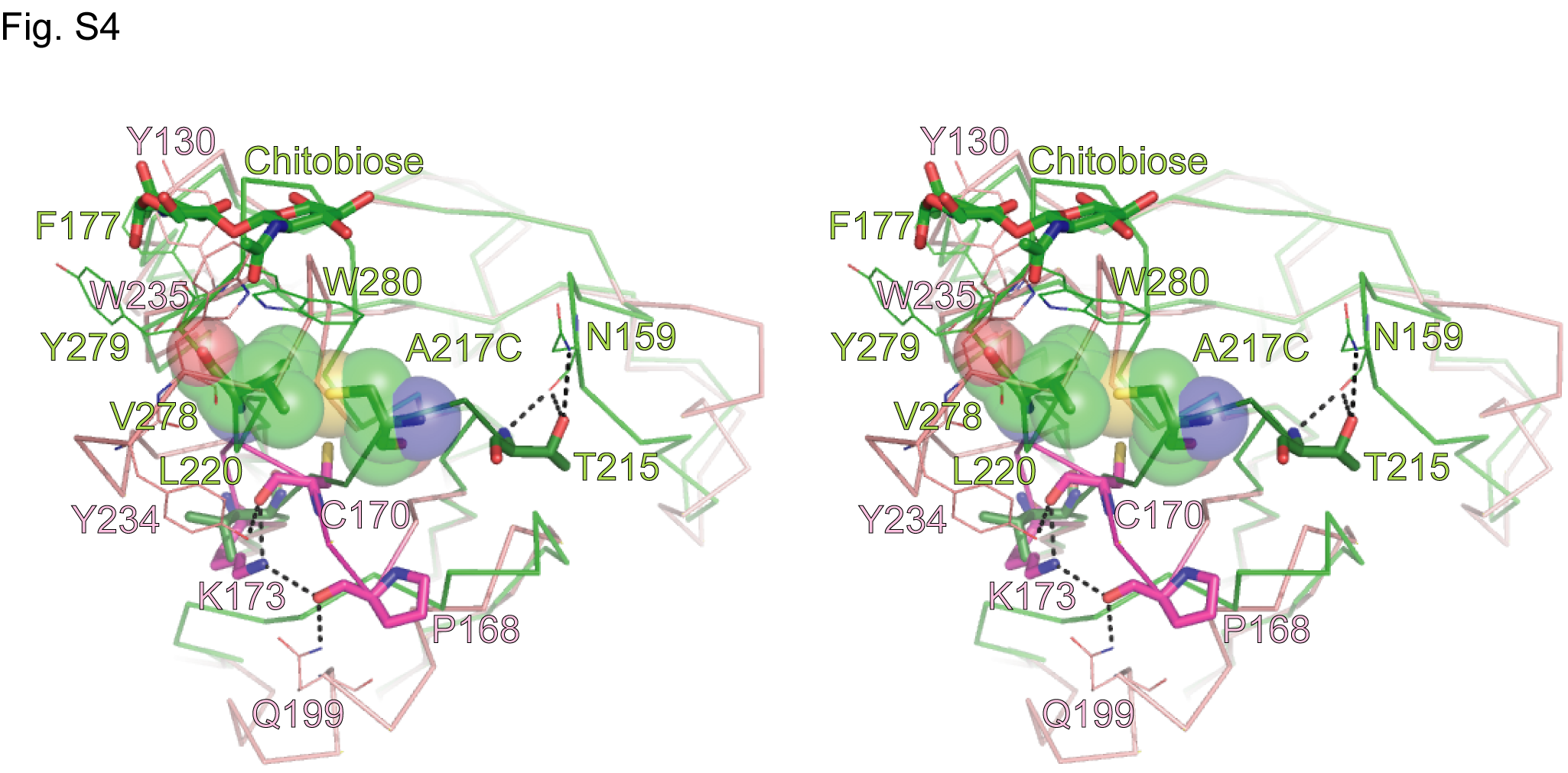

Supplement: S4 Fig — Fbs1 A217C model and FBG3 are light green and pink. Non-conserved amino acids at the loop β5-β6 are represented using stick models. Hydrogen bonds between non-conserved residues and other loops are indicated by dotted lines. A217C and V278 in Fbs1 are represented using sphere models. The sphere models show the side chain conflict between the sulfur atom (yellow sphere) of A217C model and carbon atom (green sphere) of V278. (TIF) [file pone.0140366.s004.tif]
